# Role of Endogenous Opioid System in Ischemic-Induced Late Preconditioning

**DOI:** 10.1371/journal.pone.0134283

**Published:** 2015-07-30

**Authors:** Jan Fraessdorf, Markus W. Hollmann, Iris Hanschmann, André Heinen, Nina C. Weber, Benedikt Preckel, Ragnar Huhn

**Affiliations:** 1 Department of Anesthesiology, University Hospital Duesseldorf, Duesseldorf, Germany; 2 Department of Anesthesiology, Laboratory of Experimental Intensive Care and Anesthesiology (L.E.I.C.A.), Academic Medical Center (AMC), University of Amsterdam, Amsterdam, The Netherlands; 3 Department of Cardiovasacular Physiology, Heinrich-Heine-University Duesseldorf, Duesseldorf, Germany; University of Colorado Denver, UNITED STATES

## Abstract

**Background:**

Opioid receptors (OR) are involved in myocardial late preconditioning (LPC) induced by morphine and δ1-opioid receptor (δ1-OR) agonists. The role of OR in ischemic-induced LPC is unknown. We investigated whether 1) OR are involved in the trigger and/or mediation phase of LPC and 2) a time course effect on the expression of different opioid receptors and their endogenous ligands exists.

**Methods:**

Male Wistar rats were randomly allocated to four groups (each group n = 8). Awake animals were ischemic preconditioned by a 5 minutes coronary occlusion. 24 hours later, anesthetized animals underwent 25 minutes coronary occlusion followed by 2 hours of reperfusion. The role of OR was investigated by treatment with intraperitoneal naloxone (Nal) 10 minutes prior to LPC (Nal-LPC; trigger phase) or 10 min prior to sustained ischemia (LPC-Nal; mediation phase).

**Results:**

LPC reduced infarct size from 61±10% in controls to 25±9% (P<0.001). Naloxone during trigger or mediation phase completely abolished LPC-induced cardioprotection (59±9% and 62±9%; P<0.001 vs. LPC). 8, 12 and 24 hours after the ischemic stimulus, expression of δ-OR in the heart was increased, whereas μ-opioid receptor (μ-OR) and κ-opioid receptor (κ-OR) were not. Plasma concentrations of β-endorphin and leu-enkephalin but not dynorphin were increased by LPC.

**Conclusion:**

Ischemic LPC is triggererd and mediated by OR. Expression of δ-OR and plasma levels of endogenous opioid peptides are increased after ischemic LPC.

## Introduction

Ischemic preconditioning is a strong protective mechanism of the heart in which brief exposure to ischemia/reperfusion markedly enhances the ability to withstand a subsequent ischemic injury [1]. Two phases of preconditioning exist. An early phase (early preconditioning, EPC), which develops within a few minutes and lasts for 2–3 hours, and a late phase (late preconditioning, LPC), which develops more slowly (requiring 6–12 hours) but lasts for 2–3 days [2]. Beside ischemic stimuli a variety of other stimuli that can mimic the cardioprotective effect of late ischemic preconditioning exist, e.g. volatile anesthestics [3], the noble gases xenon and helium [4, 5], opioids [6] or statins [7].

Opioid receptors (OR) are involved in the signal transduction cascade of early ischemic preconditioning. The non-specific opioid antagonist naloxone completely abrogated the cardioprotective effect of early ischemic preconditioning [8] and the receptor subtype involved is most likely the δ1-opioid receptor (δ1-OR) [9]. Little is known regarding the involvement of OR in ischemic induced late preconditioning. In contrast, several groups clearly demonstrated that opioid receptor stimulation by pharmacological agents is able to induce late preconditioning against myocardial infarction and stunning and that most likely the δ1-OR is involved. Additionally, we recently demonstrated that morphine induces late preconditioning and that this effect was abolished by naloxone during the trigger and the mediation phase. Surprisingly, not only morphine induced late cardioprotection is OR dependent, but also lipopolysaccharide induced cardioprotection is mediated, but not triggered, by opioid receptors [6]. This observation indicates a possible role of endogenous opioids in late preconditioning.

We hypothesize that in the rat heart in vivo a) OR are involved in the trigger and/or mediation phase of ischemic LPC and, b) that a time course effect in the expression of the different OR and the endogenous opioid peptides after LPC stimuli exists.

## Methods

The study was performed in accordance with the guidelines laid out in the Guide for the Care and Use of Laboratory Animals, which is available from the National Academy of Science and the regulations of the German Animal Protection Law and was approved by the District Government of Duesseldorf, Germany (G 07/04).

### Materials

Antibodies for endorphin and enkephalin were purchased from Abcam (Cambridge, UK). Antibodies for μ-OR and δ-OR were purchased from Neuromics (Minneapolis, USA), κ-OR antibody was from Abcam (Cambridge, UK). All other chemicals were purchased from Sigma-Aldrich (Taufkirchen, Germany).

### Infarct size experiments

**Fig 1:**
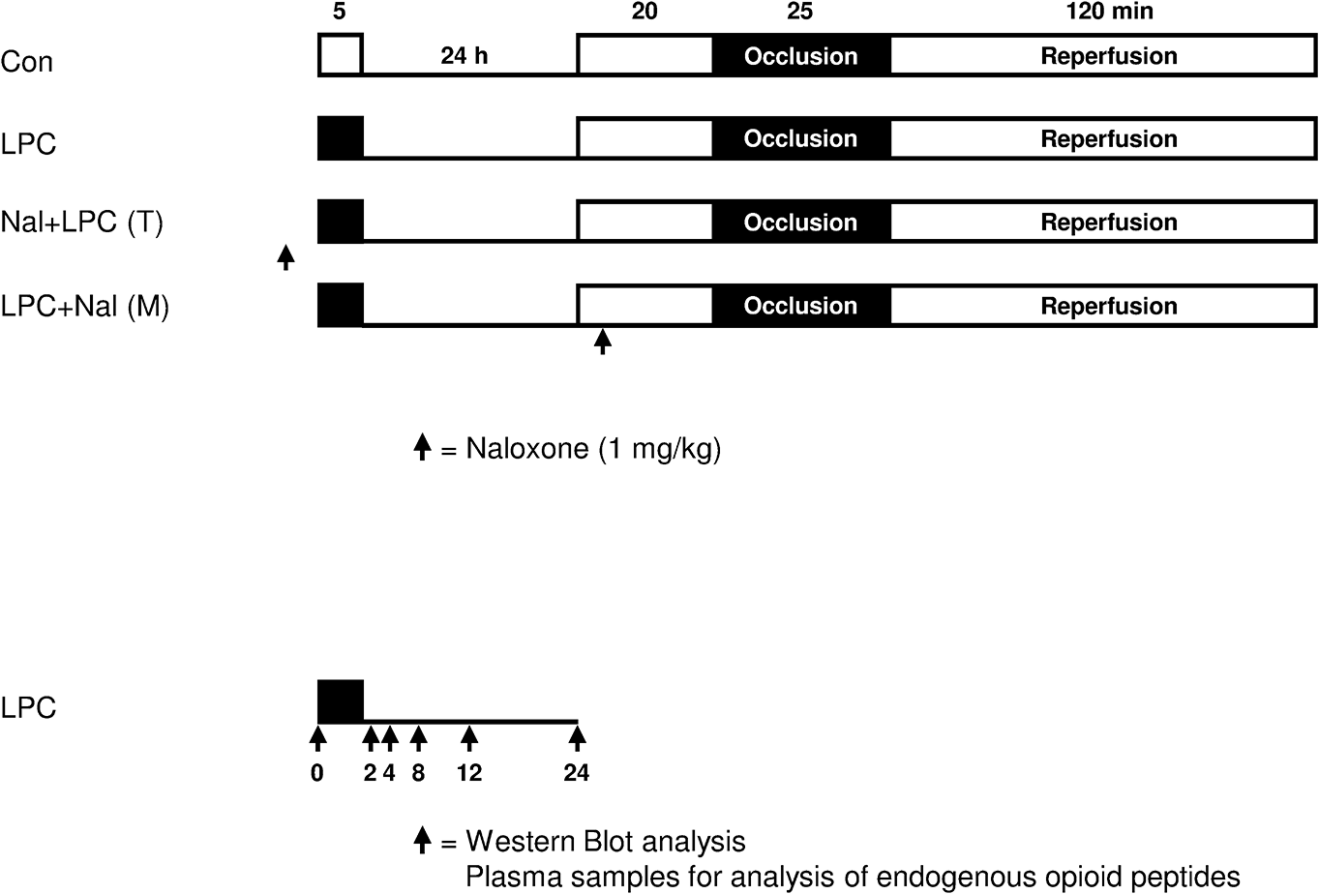
Experimental protocol for infarct size measurements and molecular biology. Con = control, LPC = late preconditioning, Nal = naloxone (1 mg/kg), (T) = trigger phase, (M) = mediation phase.

Male Wistar rats had free access to water and standard rat food at all times prior to experiments. The animal preparation was performed as described previously [5]. The animals (305±12 g) were instrumented with a coronary occluder around a left anterior descending artery. 7 days after recovery, animals were randomly allocated into four groups (by sealed envelopes; each group n = 8, Fig 1). Awake animals were ischemic preconditioned by a 5 minute coronary artery occlusion. 24 hours later, chloralose-anesthetized animals were endotracheally intubated with a plastic cannula (outer diameter 2.2 mm). After a median incision at the cervical level was performed a 20 gauge cannula was inserted into the right internal carotid artery and advanced into the aorta in order to measure aortic pressure. Aortic pressure was digitized using an analogue to digital converter (PowerLab/8SP, ADInstruments Pty Ltd, Castle Hill, Australia) at a sampling rate of 500 Hz and were continuously recorded on a personal computer using Chart for Windows v5.0 (ADInstruments Pty Ltd, Castle Hill, Australia). All animals underwent 25 minutes of coronary artery occlusion followed by 2 hours of reperfusion. At the end of reperfusion the hearts were excised and infarct sizes were determined using a previously described method [5]. Briefly, the heart was excised with the occluding suture left in place and then mounted on a modified Langendorff apparatus for perfusion with ice cold normal saline via the aortic root at a perfusion pressure of 80 cm H_2_O in order to wash out intravascular blood. After 5 minutes of perfusion, the coronary artery was re-occluded and the remainder of the myocardium was perfused through the aortic root with 0.2% Evans blue in normal saline for 10 minutes. Intravascular Evans blue was then washed out by perfusion with normal saline for 10 min. This treatment identified the area at risk as unstained. The heart was then cut into 2 mm thick transverse slices. The slices were stained with 0.75% triphenyltetrazolium chloride (TTC) solution for 10 minutes at 37°C, and fixed in 4% formalin solution for 24 hours at room temperature. The area at risk and the infarct size were determined using planimetry and corrected for dry weight in each slice by using SigmaScan Pro5 (SPSS Science Software, Chicago, IL, USA).

### Experimental protocol

To investigate the influence of the opioid receptor system on ischemic induced LPC rats were randomly (by using sealed envelopes) allocated to four groups after recovery from surgery (Fig 1). The dosage of naloxone chosen was the same as in our previous work [6] where naloxone abolished the effect of LPC without having an own effect on infarct size or on global hemodynamics.

#### Control group (Con)

24 hours prior to ischemia/reperfusion experiments, all animals (n = 8) of the Con group underwent a sham procedure (taken out of their cage without inducing ischemia/reperfusion). On the day of the final experiments rats were anesthetized and underwent the ischemia/reperfusion protocol as described above.

#### Late preconditioning group (LPC)

24 hours prior to ischemia/reperfusion experiments, all animals (n = 8) of the LPC group underwent a 5 minute period of ischemia, followed by reperfusion. Ischemia was then induced by occluding the coronary occluder for 5 minutes. On the day of the final experiments rats were anesthetized and underwent the ischemia/reperfusion protocol as described above.

#### Naloxone & LPC group (Nal+LPC); trigger phase

24 hours prior to ischemia/reperfusion experiments, all animals (n = 8) of the Nal+LPC group underwent a 5 minute period of ischemia, followed by reperfusion. Ischemia was then induced by occluding the coronary occluder for 5 minutes. 10 minutes prior to the ischemic LPC, trigger animals received 1 mg/kg naloxone hydrochloride i.p. On the day of the final experiments rats were anesthetized and underwent the ischemia/reperfusion protocol as described above.

#### LPC and naloxone group (LPC+Nal), mediation phase

24 hours prior to ischemia/reperfusion experiments, all animals (n = 8) of the LPC+Nal group underwent a 5 minute period of ischemia, followed by reperfusion. Ischemia was then induced by occluding the coronary occluder for 5 minutes. On the day of the final experiments rats were anesthetized and underwent the ischemia/reperfusion protocol as described above. 10 minutes prior to the index ischemia (in the mediation phase), animals received 1 mg/kg of naloxone hydrochloride i.p.

### Western Blot experiments

To investigate the expression of δ-OR, κ-OR and μ-OR in ischemic-induced LPC, additional experiments were conducted in 42 rats (7 groups, n = 6 per group). In order to determine the time-course of the expression of δ-OR, κ-OR and μ-OR after 5 min of awake coronary occlusion, animals were sacrificed at several, for LPC relevant time points (Sham, 0, 2, 4, 8, 12 and 24 hours, each time point n = 6, Fig 1) and myocardial samples were taken from the area at risk (= area that has been ischemic).

### Endogenous opioid peptides analysis

Additional experiments were conducted to investigate the time course effect of β-endorphin, dynorphin and leu-enkephalin. We measured at the same time points as OR in plasma samples (Sham, 0, 2, 4, 8, 12 and 24 hours, each time point n = 4, Fig 1). Blood was sampled, processed and stored according to the manual of the respective enzyme immune assay kits (Endorphin-beta, dynorphin A and enkephaline-leucin; Phoenix Pharmaceutical Inc., Burlingame, CA, USA).

In brief, samples were collected and centrifuged to obtain plasma specimen. Plasma proteins were extracted using commercial available separation columns (SEP-C18 column, Phoenix Pharmaceuticals) and evaporated to dryness and further processed according the manufacturer assay protocol.

### Western Blot analysis

Analysis of the OR by Western Blot was performed in whole tissue lysates as described previously [10].

### Statistical analysis

Data are expressed as mean±SD. Sample size analysis revealed that a group size of n = 8 was necessary to detect a difference in infarct size of 30% with a power of 95% and an α of 0.05 (two-tailed). For Western Blot analysis a group size of n = 6 was necessary to detect a difference between the groups of 30% with a power of 95%, a standard deviation of 10 and an α of 0.05 (two-tailed). Statistical analysis was performed by ANOVA with Bonferroni's multiple comparison test (two-tailed, Graph Pad Prism, v4.00, Graph Pad Software, La Jolla, Ca). Statistical analysis of the hemodynamic variables was performed by two-way ANOVA for time and treatment effects. If an overall significance was found, comparisons between groups were made for each time point using ANOVA followed by Dunnett post hoc test with the control group as reference group. Time effects within each group were analyzed by repeated measures ANOVA followed by two-tailed Dunnett post hoc test with the baseline value as the reference time point. P<0.05 was considered statistically significant.

## Results

### Infarct size measurement

**Fig 2:**
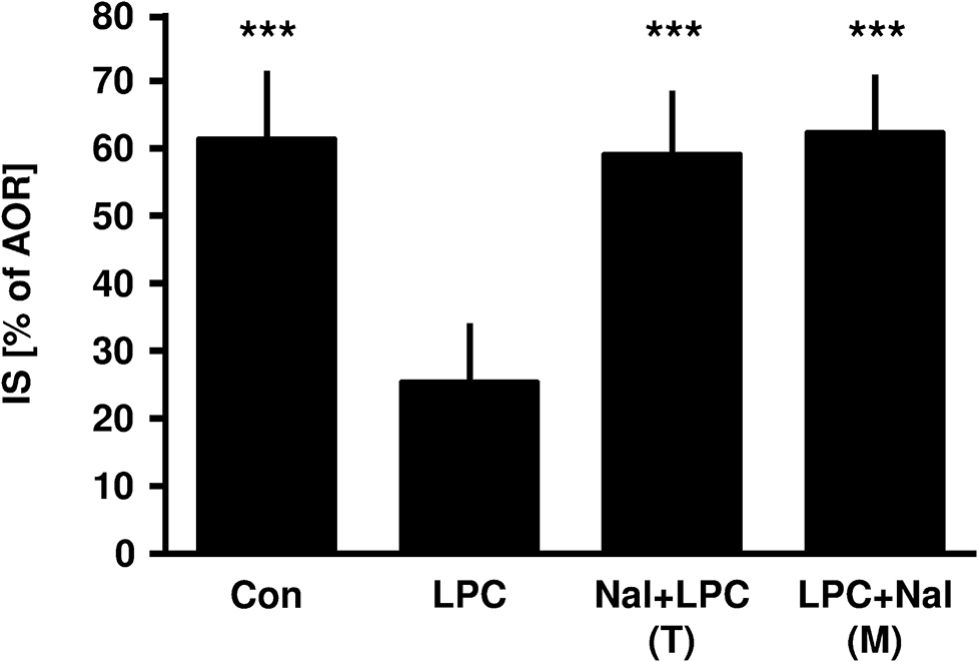
Infarct size measurement. Histogram shows the infarct size (percent of area at risk) of controls (Con), ischemic late preconditioning (LPC), naloxone (1 mg/kg) in the trigger phase combined with ischemic late preconditioning (Nal+LPC) and naloxone (1 mg/kg) in the mediation phase combined with ischemic late preconditioning (LPC+Nal). Data are presented as mean±SD, ***P<0.001 vs. LPC group.

Ischemic LPC led to an infarct size reduction compared to control animals (LPC: 25±9% vs. Con: 61±10%, P<0.001; Fig 2). The opioid antagonist naloxone abolished this cardioprotective effect in the trigger (Nal+LPC: 59±9%, P<0.001 vs. LPC) as well as in the mediation phase (LPC+Nal: 62±9%, P<0.001 vs. LPC). We could previously show in the same animal model that naloxone itself had no effect on infarct size in the trigger or in the mediation phase, respectively [6].

### Hemodynamic measurements

No differences in heart rate and mean arterial pressure were observed between the groups during baseline (Table 1). Baseline 1 is the time point after recovery from the surgical procedure; baseline 2 is the time point after administration of naloxone in the LPC+Nal group and vehicle in the corresponding groups respectively (Fig 1). At the end of reperfusion heart rate was significantly lower in the control group compared to the ischemic LPC groups. Furthermore, mean aortic pressure was significantly decreased in the Nal+LPC and LPC+Nal groups compared to control and LPC groups, but in all four groups mean aortic pressure was lower compared to baseline values.

**Table 1:** Hemodynamic variables.

	Baseline 1	Baseline 2	Ischemia		Reperfusion	
			5	24	30	60
Heart Rate (bpm)						
Con	414±8	414±10	391±19	382±24^†††^	392±18	366±7^†††^
LPC	417±17	422±15	399±24	399±24	405±14	397±14**
Nal+LPC	418±14	426±18	415±13	409±16*	420±16*	399±18**
LPC+Nal	409±16	419±18	422±18**	412±26*	421±21*	392±25*
Mean Aortic Pressure (mmHg)						
Con	109±11	113±5	100±8	85±12^†††^	85±8^†††^	84±8^†††^
LPC	119±8	121±11	99±13	100±12	93±15^†^	91±24^†^
Nal+LPC	114±14	121±8	91±12	80±39^†^	71±19^†††^	56±27* ^†††^
LPC+Nal	119±7	117±12	90±19^†^	84±20^†††^	78±19^†††^	57±17* ^†††^

Data are mean±SD.

Con = control; LPC = late ischemic preconditioning; Nal+LPC = naloxone administration prior to late ischemic preconditioning (trigger phase); LPC+Nal = naloxone administration prior to index ischemia (mediation phase).

*p<0.05 vs. Con

** p<0.01 vs. Con

^†^p<0.05 vs. Baseline

^†††^ p<0.001 vs Baseline.

### Time course expression of opioid receptors

**Fig 3:**
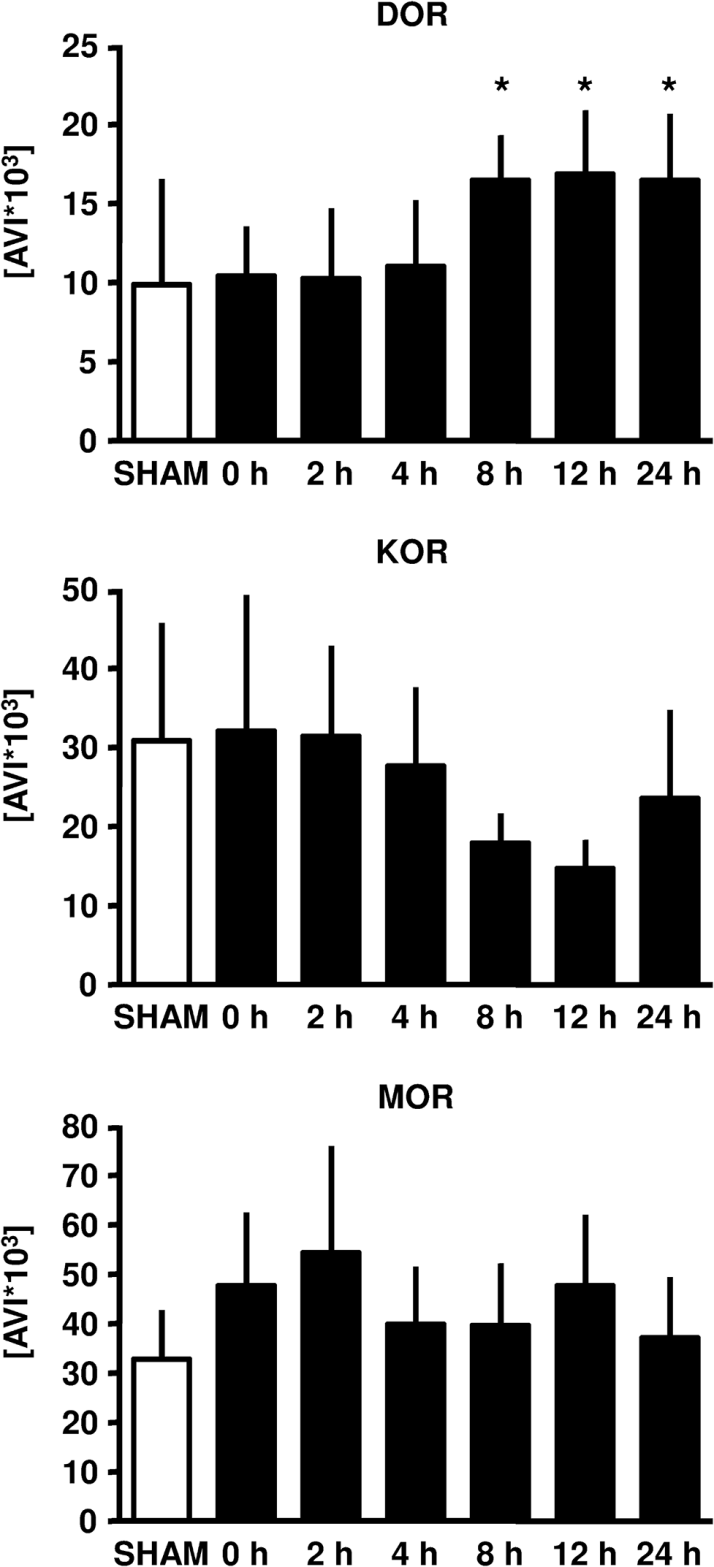
DOR, KOR and MOR expression in rat myocardium upon ischemic late preconditioning (LPC). Representative western blot analysis experiments of a time course (Sham, 0, 2, 4, 8, 12 and 24 hours after the ischemic stimulus was initiated). Summarized data presenting AVI (arbitray units of average light intensity) are shown. Data are presented as mean±SD, *P<0.05 vs. 0 hours.

8 hours following ischemic LPC, expression of δ-OR was increased and expression remained elevated up to 24 hours in myocardial samples (Fig 3). We could not observe any changes in the expression of κ-OR or μ-OR at any of the measured time points (Fig 3).

### Time course of plasma concentrations of endogenous opioid peptides

**Fig 4:**
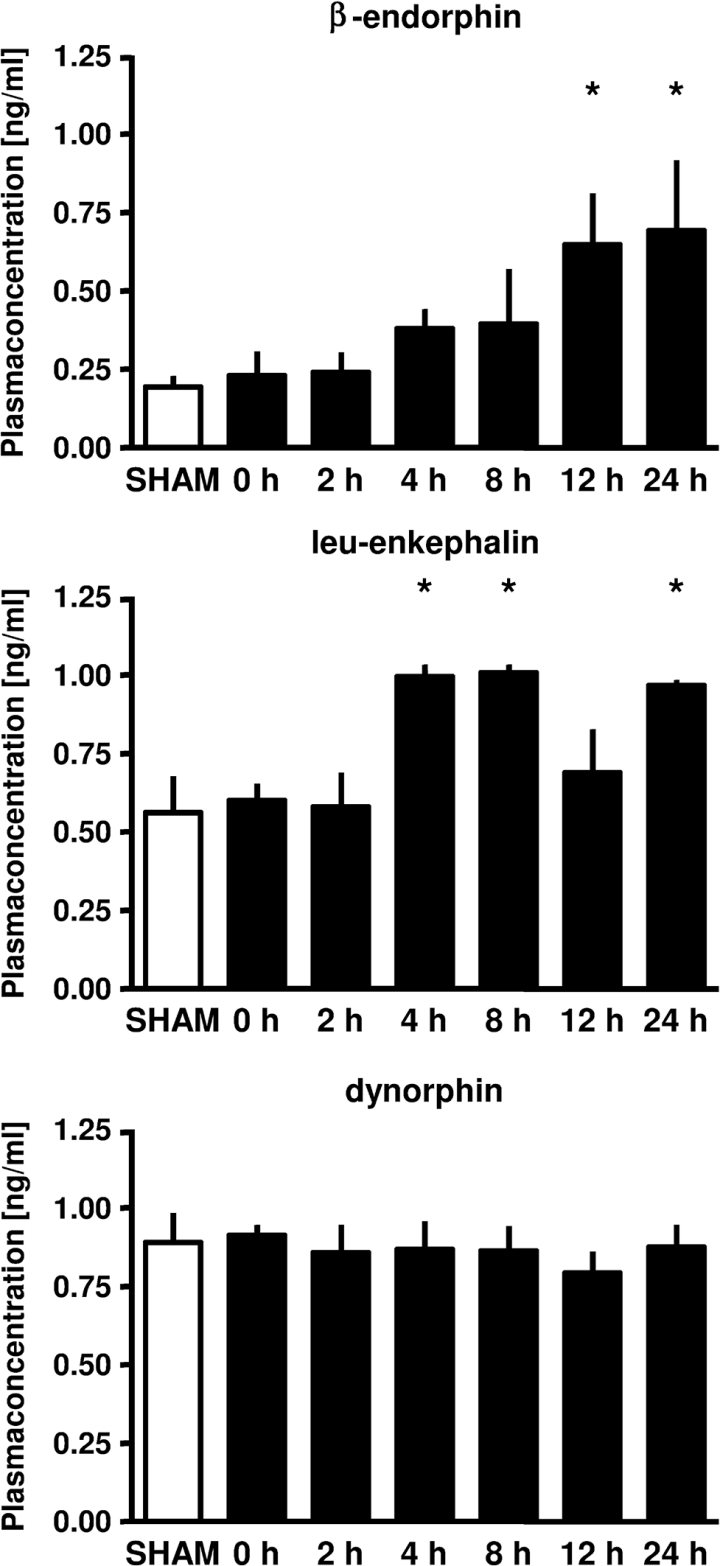
Plasma concentrations (ng/ml) of the endogenous opioids β-endorphin, dynorphin and leu-enkephalin in rat myocardium upon ischemic LPC (Sham, 0, 2, 4, 8, 12 and 24 hours after the ischemic stimulus was initiated). Data are presented as mean±SD, *P<0.05 vs. 0 hours.

12 and 24 hours after the preconditioning stimulus, plasma concentrations of β-endorphin were increased (12h: 0.65±0.16 ng/ml, 24h: 0.69±0.22 ng/ml; each P<0.01 vs. Con: 0.23±0.08 ng/ml, Fig 4). Plasma concentrations of leu-enkephalin were elevated 4, 8 and 24 hours after ischemic LPC (4h: 1.00±0.04 ng/ml, 8h: 1.01±0.03 ng/ml, 24h: 0.97±0.02 ng/ml; each P<0.01 vs. Con: 0.60±0.05 ng/ml, Fig 4). There were no changes in dynorphin plasma concentrations at all measured time points (Fig 4).

## Discussion

In the present study we demonstrate that 5 minutes of coronary artery occlusion in the awake rat 24 hours before index ischemia induces late preconditioning of the heart *in vivo* as measured by reduced infarct size compared to non-preconditioned animals. Kloner et al. could show that a previous angina before myocardial infarction confers a beneficial effect on in-hospital outcome possibly through the mechanism of ischemic preconditioning [11]. Baxter et al. showed that more than one preconditioning cycle on the same day 24 hours before ischemia and reperfusion did not enhance the infarct size limiting effect of ischemic LPC [12]. So we assume that one 5 minute cycle of coronary artery occlusion induces the maximal cardioprotective effect in terms of LPC.

This is the first study demonstrating an increased expression of δ-ORs after ischemic-induced LPC. By means of pharmacological blockade we demonstrated that opioid receptors are involved during trigger and mediation phase. Our data indicate which subtype of OR is involved in ischemic induced LPC. Studies looking at *early* ischemic induced preconditioninig have shown that δ-OR and κ-OR subtypes are involved, whereas all three OR subtypes are involved in pharmacologically- induced EPC [13]. δ-OR [14] and κ-OR [15] agonists are able to induce LPC. However, we now report for the first time on the role of OR in ischemic induced LPC.

There is conflicting evidence regarding the expression of different OR subtypes in the myocardium. Witter et al. reported in 1996 that only δ-OR and κ-OR are expressed in myocardium of Sprague Dawley rats, whereas they could not detect any μ-OR receptor mRNA in the myocardium. In contrast, Bell et al. demonstrated predominantly μ-OR and δ-OR expression in human right ventricular probes [16]. However, we were able to demonstrate in samples taken from the whole heart (Wistar rats) that all three OR subtypes are expressed. This is the first study demonstrating also the presence of μ-OR`s in rat myocardium. Whether these conflicting results are due to strain differences remain unclear. Alternatively, since we performed our Western blot analysis in whole heart samples, we might have measured OR that are present in blood born cells (i.e. lymphocytes).

In the present study we induced preconditioning by a 5 minute ischemic stimulus in the awake animal. We focused on a possible time effect for regulation of the different OR and endogenous opioid peptides which has not been investigated so far. Our results demonstrate a time-dependent increased expression of δ-OR. As we performed Western Blot in whole cell lysate these findings do not reflect shifts between cellular compartements but altered myocardial expression. Interestingly, we found a time-dependent increase in β-endorphin and leu-enkephalin, two endogenous opioid peptides with the same affinity to μ-OR and δ-OR. It is conceivable that a 5 minute ischemic stimulus is sufficient to induce an infarct size limiting effect, but is not strong enough to increase the expression of μ-OR. A second or longer ischemic stimulus might have led to an increased expression of μ-OR too. The opioid morphine, known as a μ-OR agonist with a low affinity to δ-OR and κ-OR [17] has been shown to induce a delayed cardioprotective effect [6, 18, 19, 20]. Additionally, the more μ-OR specific opioid remifentanil also induces LPC in a μ-OR dependent manner [13].

We measured plasma concentrations of the endogenous opioid peptides β-endorphin, dynorphin and leu-enkephalin at the same time points as we investigated the expression of OR. The heart is able to synthesize and to store all three endogenous opioid peptides [21, 22] and these peptides are released during cellular stress [23, 24]. Jackson et al. demonstrated an increase in enkephalin concentration in the sinus node during short terms of ischemia, returning back to baseline during reperfusion [25]. Paradis et al. [26] observed an increase in enkephalin concentration in rat hearts during ischemia. Gao et al. reported that δ-OR and Leu-enkephaline are up-regulated and involved in hypoxia-induced LPC following global cerebral ischemia in rats [27]. These observations, together with the fact that ischemic induced preconditioning is abolished by blockade of ORs suggest that endogenous opioid peptides serve as an autocrine mediator during ischemia and reperfusion. We could not detect any effect by ischemic preconditioning on dynorphin plasma concentrations at all measured time points. However, we demonstrated an increase in plasma concentrations of β-endorphin and leu-enkephalin by a 5 minute ischemic stimulus. β-endorphin levels were increased 12 hours after the ischemic stimulus and remained elevated until 24 hours. Plasma concentrations of leu-enkephalin were increased after 4, 8 and 24 hours, but not 12 hours following the preconditioning stimulus. β-endorphin and leu-enkephalin act on the δ-OR and the μ-OR. This is consistent with our results regarding the expression of δ-OR´s. We only detected an increase in δ-OR expression after 8, 12 and 24 hours. In contrast, the endogenous opioid peptide dynorphin acts on κ-OR´s and here we could not detect any time dependent difference in both, dynorphin plasma concentrations and κ-OR expression. It remains unclear whether the increase in plasma level of the opioid peptides induces the enhanced δ-OR expression. Neuronal in vitro preconditioning studies revealed increased δ-OR expression after a preconditioning like stimulus (hypoxia), whereas leu-enkephalin remained stable. These data indicate that the expression of δ-OR seems to be independent of the endogenous ligand [28]. In isolated myocardial nuclei from cardiomyopathic hamsters exposure to an endogenous opioid recpetor ligand (dynorphin B) markedly increased opioid peptide gene transcription [29]. As almost nothing is known how OR expression is regulated in the myocardium it remains elusive if increased expression of OR is leading to increased plasma levels of endogenous opioid peptides or vice versa.

Thus, results from OR expression and endogenous opioid peptide plasma concentrations suggest that ischemic-induced LPC protects the heart via δ-OR activation with concomitant increase of the endogenous opioid peptides β-endorphin and leu-enkephalin.

### Limitations of the study

We did not confirm our results from molecular biology with the opioid antagonist naloxone as we did in infarct size experiments. However, our results from infarct size experiments clearly demonstrate that cardioprotection is abolished when naloxone is given in the trigger and mediation phase. Compared with controls we observed a decreased mean aortic pressure in naloxone treated animals at the end of reperfusion (Table 1). Based on the results of our previous study [6] direct hemodynamic effects of naloxone in our experimental setting can be ruled out. As the decrease in mean aortic pressure occurs only at the end of reperfusion, any influence on our findings seem to be highly unlikely.

We can not rule out that expression of δ-OR´s is also increased in the presence of naloxone and it might be possible that only the cardioprotective effect is blocked at the level of the receptor and can not be transferred to a cellular response. Second, we did not investigate possible intracellular candidates of late preconditioning like COX-2 (cyclooxygenase-2) and/or iNOS (inucible nitric oxide synthase). Guo et al. could show that the cardioprotective effect of LPC induced by δ-OR activation is mediated by iNOS [30]. The same author showed that also COX-2 is critically involved in ischemic-induced LPC [31]. In the present study, we were interested in the involvement of the endogenous opioid system in the trigger- and/or mediation phase of ischemic-induced LPC and thus investigations of intracellular enzymes was beyond the scope of our study.

In conclusion our results indicate, for the first time, that increased expression of OR, especially δ-OR’s, is associated with ischemic LPC. Furthermore, the activation of the endogenous opioid system is required in the trigger and mediation phase of LPC.
